# Five-Year Follow-Up for Women With Subclinical Hypothyroidism in Pregnancy

**DOI:** 10.1210/jc.2013-2768

**Published:** 2013-11-11

**Authors:** Beverley M. Shields, Bridget A. Knight, Anita V. Hill, Andrew T. Hattersley, Bijay Vaidya

**Affiliations:** National Institute for Health Research Exeter Clinical Research Facility (B.M.S., B.A.K., A.V.H., A.T.H., B.V.), University of Exeter Medical School, University of Exeter, Exeter, United Kingdom; and Department of Endocrinology (B.A.K., B.V.), Royal Devon and Exeter NHS Foundation Trust, Exeter, United Kingdom

## Abstract

**Context:**

Increasing numbers of women are being treated with l-thyroxine in pregnancy for mild thyroid dysfunction because of its association with impaired neuropsychological development in their offspring and other adverse obstetric outcomes. However, there are limited data to indicate whether treatment should be continued outside of pregnancy.

**Objectives:**

We aimed to determine whether subclinical hypothyroidism and maternal hypothyroxinemia resolve postdelivery.

**Design, Setting, and Participants:**

A total of 523 pregnant healthy women with no known thyroid disorders were recruited during routine antenatal care and provided blood samples at 28 weeks of pregnancy and at a mean of 4.9 years postpregnancy.

**Main Outcome Measures:**

TSH, free T_4_, free T_3_, and thyroid peroxidase antibody levels were measured in serum taken in pregnancy and at follow-up.

**Results:**

Subclinical hypothyroidism in pregnancy (TSH >3 mIU/L) was present in 65 of 523 (12.4%) women. Of these, 49 (75.4%) women had normal thyroid function postpregnancy; 16 of 65 (24.6%) had persistent high TSH (TSH >4.5 mIU/L postpregnancy) with 3 women receiving l-thyroxine treatment. A total of 44 of 523 (8.4%) women had isolated maternal hypothyroxinemia in pregnancy (free T_4_ <10th centile and TSH ≤3 mIU/L). Only 2 of 44 (4.5%) had TSH >4.5 mIU/L outside pregnancy. Of the women with subclinical hypothyroidism in pregnancy with antibody measurements available, those with thyroid peroxidase antibodies in pregnancy were more likely to have persistently elevated TSH or be receiving l-thyroxine replacement after pregnancy (6 of 7 [86%] vs 10 of 57 [18%], *P* < .001).

**Conclusions:**

The majority of cases of subclinical hypothyroidism in pregnancy are transient, so treatment with l-thyroxine in these patients should be reviewed because it may not be warranted after pregnancy.

Increasing numbers of clinicians and hospitals are testing thyroid function in pregnancy to detect and treat mild thyroid dysfunction (1, 2). The use of trimester-specific reference ranges in routine clinical practice results in milder forms of thyroid dysfunction (subclinical hypothyroidism and isolated maternal hypothyroxinemia) being diagnosed in as many as 15% of pregnant women (3, 4).

Mild thyroid dysfunction has been associated with impaired neuropsychological development of the offspring and adverse obstetric outcomes including miscarriage, premature birth, gestational hypertension, and neonatal death (5–11). There is a general consensus that subclinical hypothyroidism detected during pregnancy should be treated with l-thyroxine, particularly in the presence of thyroid peroxidase antibodies (TPO-Abs) (12–14). The recent guidelines from The Endocrine Society recommend l-thyroxine replacement in all pregnant women with subclinical hypothyroidism (12); the American Thyroid Association guidelines also recommend l-thyroxine for pregnant women with subclinical hypothyroidism and positive results for TPO-Abs (13). Furthermore, contrary to the American Thyroid Association guidelines (13), a recent survey has shown that 40% of European endocrinologists also treat maternal hypothyroxinemia with l-thyroxine (1).

There are no data to indicate whether the treatment for these conditions should be limited only to during the pregnancy or continued long-term, and no advice is provided in the current guidelines (12–14). Physiological changes during pregnancy (for example, increased renal excretion of thyroxine, transfer of thyroxine to the fetus, and breakdown of thyroxine by placental deiodinases) affect thyroid economy, predisposing a woman to thyroid deficiency (6), so it is likely that these effects are transient.

We aimed to study the natural history of mild thyroid hormone deficiency detected during pregnancy and hypothesized that most cases of subclinical hypothyroidism and maternal hypothyroxinemia resolve postdelivery, thus providing evidence that women being treated for these conditions may not need to continue receiving long-term l-thyroxine replacement postpregnancy.

## Materials and Methods

### Subjects

A total of 988 pregnant healthy women were recruited as part of the Exeter Family Study of Childhood Health between 1999 and 2004. A detailed protocol of this study and background data on the participants were published previously (15). Blood samples were taken at 28 weeks of pregnancy. Thyroid function tests (TSH, free T4 [FT_4_] and free T_3_ [FT_3_] levels) were performed on the stored serum samples, and the presence of TPO-Abs was determined. Of the recruited patients, 32 were excluded: 21 were taking thyroid-related medications (18 taking l-thyroxine and 3 taking propylthiouracil), 10 had overt hypothyroidism (TSH >4.5 mIU/L and FT_4_ <11 pmol/L), and 1 had overt hyperthyroidism (TSH <0.01 mIU/L and FT_4_ >24 pmol/L or FT_3_ >6.8 pmol/L). Therefore, 956 women were suitable for analysis.

All women were invited for a follow-up study taking repeat measurements outside pregnancy, and 523 of these women took part and were included even if they were now taking l-thyroxine. The same thyroid function tests were performed at the postpregnancy visit 4.9 ± 1.6 years (mean ± SD) after delivery. The 523 women who had follow-up data were slightly older (median 32 vs 30 years, *P* < .001), slimmer (prepregnancy body mass index 22.8 vs 23.6 kg/m^2^, *P* = .03), and less likely to smoke during pregnancy (51 of 523 [10%] vs 80 of 429 [19%], *P* < .001) compared with the 433 women who did not have follow-up data. Pregnancy TSH and FT_4_ results were similar between those who had follow-up results and those who did not (median TSH: 1.87 vs 1.8 mIU/L, *P* = .8; median FT_4_: 12.00 vs 12.08 pmol/L, *P* = .6), but FT_3_ results were marginally higher in those not recruited at follow-up (median 4.16 vs 4.13 pmol/L, *P* = .009). Some patients had missing data for smoking and subsequent pregnancies at the follow-up visit, so the numbers are smaller for some analyses (see Table 1).

**Table 1.** Demographic Data and Thyroid Function Test Results for 523 Women at 28 Weeks of Pregnancy and 4.9 ± 1.6 Years After Pregnancy

	During Pregnancy	Postpregnancy	*P*^a^
Subject characteristics			
Age, y	32 (29–35)	37 (33–40)	
Body mass index, kg/m^2^	26.5 (24.4–29.7)	23.7 (21.8–26.8)	<.001
Smoking^b^	46/454 (10)	62/454 (14)	.009
Pregnancy details			
Primipara	236 (45)		
Subsequent pregnancies (n = 521^b^)			
0		294	
1		207	
2		18	
>2		2	
Cesarean delivery^b,c^	108/520 (21)	55/220 (25)	
Thyroid function tests and TPO-Abs			
TSH, mIU/L	1.81 (1.37–2.45)	1.89 (1.32–2.63)	.01
FT_4_, pmol/L	12.1 (11.2–13.0)	14.8 (13.6–16.0)	<.001
FT_3_, pmol/L	4.1 (3.9–4.4)	4.5 (4.2–4.8)	<.001
TPO-Ab positive^b^	27/520 (5.1)	64/520 (12.3)	<.001
Receiving l-thyroxine	NA	8/523 (1.5)	

Abbreviation: NA, not applicable. Data are presented as median (interquartile range) or n (%).

a *P* values were determined by the Wilcoxon or McNemar test for related data and are not presented for age as all patients were older postpregnancy.

b Some missing data.

c Postpregnancy number represents any women who had a cesarean delivery in a subsequent pregnancy.

All subjects gave informed consent, and ethical approval was obtained from the North and East Devon Local Research Ethics Committee

### Analysis of Thyroid Function and TPO-Abs

Serum TSH, FT_4_, and FT_3_ were analyzed using an electrochemiluminescent immunoassay, run on the modular E170 analyzer (Roche). Intra-assay coefficients of variation were as follows: TSH, <5.3%; FT_4_, < 5.3%; and FT_3_, <5.1%. TPO-Abs were analyzed using a competitive immunoassay (Roche), and a titer >34 IU/mL was considered positive. High TSH in pregnancy was defined as >3 mIU/L, the generally accepted upper limit of the reference range in the second and third trimesters (12, 13). High TSH outside of pregnancy was defined as >4.5 mIU/L, the upper limit of the manufacturer's population reference range (reference ranges: TSH, 0.35–4.5 mIU/L; FT_4_, 11–24 pmol/L; and FT_3_, 3.9–6.8 pmol/L). TPO-Ab results were missing for 3 patients.

### Statistical Analysis

Thyroid function test results were not normally distributed, and even with log transformations, normality could not be achieved because of outliers. Therefore, nonparametric statistics were used to analyze these data. For comparisons between pregnancy and postpregnancy measurements, Wilcoxon (for continuous data) and McNemar (for discrete data) tests for paired analysis were used. For assessment of differences between those who were recruited at follow-up and those who were not, the Mann-Whitney *U* test was used.

## Results

### Study population

Table 1 shows the demographic data and thyroid function results of the 523 women in this study cohort. TSH, FT_4_, and FT_3_ levels were higher when measured at 5 years postpregnancy than for the same women in pregnancy.

### Follow-up thyroid function tests in women with subclinical hypothyroidism in pregnancy

**Figure 1.**
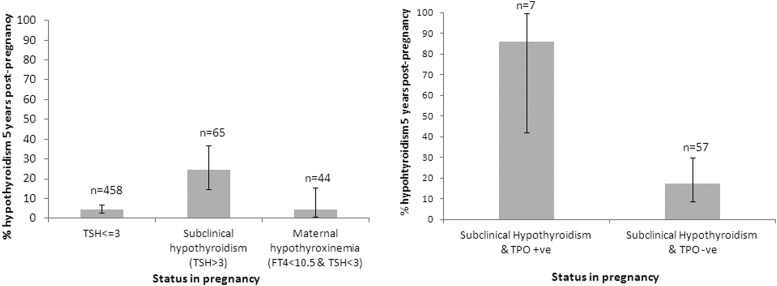
Prevalence of hypothyroidism 5 years postpregnancy. Bar charts show the prevalence of hypothyroidism 5 years postpregnancy in (A) women with TSH ≤3 mIU/L, subclinical hypothyroidism (TSH >3 mIU/L), or maternal hypothyroxinemia in pregnancy (FT4 <10.5 pmol/L and TSH ≤3 mIU/L) (*P* < .0001 (by χ^2^ test) for comparison between those with TSH ≤3 mIU/L and TSH >3 mIU/L) and (B) women with subclinical hypothyroidism in pregnancy with (+ve) and without (-ve) TPO-Abs (*P* < .001). Error bars represent 95% confidence intervals. One TPO-Ab result for a patient with subclinical hypothyroidism was unavailable.

In pregnancy, 65 of 523 (12.4%) women had subclinical hypothyroidism (defined as TSH >3 mIU/L) (12, 13). At the 5-year follow-up, 49 of 65 (75.4%) women with subclinical hypothyroidism in pregnancy had normal thyroid function postpregnancy; 16 (24.6%) had elevated serum TSH on follow-up (either TSH >4.5 mIU/L [n = 13] or were treated with l-thyroxine for diagnosed hypothyroidism [n = 3]) (Figure 1A). None of these 16 women had FT_4_ <11 pmol/L postpregnancy. Thyroid function test results for those with and without subclinical hypothyroidism in pregnancy are presented in Table 2. Of the 65 women with subclinical hypothyroidism in pregnancy, those with TPO-Abs or TSH >5 mIU/L in pregnancy were more likely to have persistently elevated TSH or be receiving l-thyroxine replacement outside pregnancy (6 of 7 [86%] TPO-Ab positive vs 10 of 57 [18%] TPO-Ab negative, *P* < .001 (1 result missing); 5 of 9[56%] with TSH >5 mIU/L vs 11 of 56[20%] with TSH ≤5 mIU/L, *P* = .02) (Figure 1B). There was no difference in the rates of Cesarean sections or small for gestational age infants in those who had persistent hypothyroidism compared with those who did not (cesarean: 4 of 16 vs 12 of 49, *P* = .97; small for gestational age infants: 2 of 16 vs 5 of 49, *P* = .8). None of the 65 women with subclinical hypothyroidism in pregnancy had a preterm delivery or fetal or neonatal death in that pregnancy.

**Table 2.** Thyroid Function Test Results for Women at 28 Weeks of Pregnancy Split by Whether They Had Subclinical Hypothyroidism in Pregnancy (TSH >3 mIU/L) or Not (TSH ≤3 mIU/L)

	Pregnancy		*P*	Postpregnancy		*P*
	TSH ≤3 mIU/L (n = 458)	TSH >3 mIU/L (n = 65)		TSH ≤4.5 mIU/L (n = 486)	TSH >4.5 mIU/L (n = 28)	
TSH, mIU/L	1.7 (1.32, 2.15)	3.57 (3.24, 4.23)	<.0001	1.84 (1.28, 2.44)	5.35 (4.76, 6.77)	<.0001
FT_4_, pmol/L	12.13 (11.16, 13.06)	11.9 (11.18, 12.64)	.29	14.8 (13.6, 16)	14.0 (12.45, 15.25)	.02
FT_3_, pmol/L	4.12 (3.85, 4.42)	4.16 (3.88, 4.44)	.52	4.5 (4.2, 4.8)	4.45 (4.1, 4.8)	.67
TPO-Ab positive	48/458 (10)	16/65 (25)	.001	47/486 (10)	11/28 (39)	<.0001

Similar results were presented for postpregnancy thyroid function test results (split by women with TSH >4.5 mIU/L or with TSH≤4.5 mIU/L), excluding those taking l-thyroxine and those with Graves disease (n = 9). Data are presented as median (interquartile range) or n (%).

On the 458 women with TSH ≤3 mIU/L in pregnancy, 21 (4.6%) had biochemical hypothyroidism on follow-up (TSH >4.5 mIU/L) (Figure 1A), 5 of whom were treated with l-thyroxine. One woman had diagnosed Graves disease. Women with TSH ≤3 mIU/L with TPO-Abs in pregnancy (n = 20) were more likely to have high TSH postpregnancy than those without TPO-Abs (6 of 20[30%] vs 15 of 436[3.4%], *P* < .001).

### Follow-up thyroid function tests in women with isolated maternal hypothyroxinemia in pregnancy

Of the patients, 44 of 523 (8.4%) had isolated maternal hypothyroxinemia in pregnancy (defined as FT_4_ below the 10th centile [<10.5 pmol/L] without raised TSH [<3 mIU/L]). Only 2 of 44 (4.5%) had TSH >4.5 mIU/L outside pregnancy, both of which were TPO-Ab positive. This proportion with raised TSH levels is similar to the proportion with normal thyroid function (TSH <3 mIU/L and FT_4_ ≥10.5 pmol/L) in pregnancy (19 of 414 [.6%], *P* = .6 by the Fisher exact test).

### TPO-Abs in pregnancy

TPO-Abs in pregnancy were found in 27 of 520 (5.2%) women: of these, 12 of 27 (44%) had TSH >4.5 mU/L postpregnancy. The prevalence of TPO-Abs more than doubled postpregnancy (64 of 520 [12%] TPO-Ab positive) (Table 1). Of the women who had TPO-Abs in pregnancy, 26 of 27 (96%) had persistent TPO-Abs postpregnancy.

## Discussion

We have performed the first systematic follow-up study of subclinical hypothyroidism in pregnancy. We found that the majority (75%) of women identified as having subclinical hypothyroidism in pregnancy had normal TSH levels after pregnancy, and only 2 of 44 (4.4%) women with maternal hypothyroxinemia had high TSH levels outside pregnancy. The biggest risk factor for raised TSH levels after pregnancy was the presence of TPO-Abs. A TSH level >5 mIU/L in pregnancy was also a risk factor but not a history of cesarean section.

Increasing numbers of pregnant women with milder forms of thyroid dysfunction are being treated with l-thyroxine, and we have demonstrated that many of these women are likely to be able to stop l-thyroxine after pregnancy. Our findings suggest that if l-thyroxine is prescribed for subclinical hypothyroidism, thyroid function should be reassessed after pregnancy to confirm whether continuation of l-thyroxine is necessary. For women with a prepregnant diagnosis of hypothyroidism, for whom increased doses of l-thyroxine are required during pregnancy, current guidelines (12, 13) suggest reducing the doses of l-thyroxine to the prepregnancy dose immediately after pregnancy and reassessing thyroid function about 6 weeks later, so this would seem a reasonable point for reassessment in women with subclinical hypothyroidism diagnosed and treated during pregnancy. Because the women who develop subclinical hypothyroidism during pregnancy and discontinue l-thyroxine treatment postpartum are at risk of recurrence of the condition in subsequent pregnancies, it would be sensible for these women to have thyroid function tests before trying for a subsequent pregnancy or as soon as the pregnancy is confirmed. Women with maternal hypothyroxinemia diagnosed in pregnancy showed no greater prevalence of hypothyroidism postpregnancy than those with normal thyroid function; therefore, follow-up of these patients outside of pregnancy may not be needed. It is also possible that maternal hypothyroxinemia may merely represent the physiological decrease in FT_4_ values with progression of pregnancy. Previously, Haddow et al (5) found that 64% of the women with undiagnosed mild maternal hypothyroidism during pregnancy had a confirmed diagnosis of overt hypothyroidism on follow-up >10 years later. However, their cohort had much higher TSH levels (mean 13.2 mIU/L) than pregnant women with subclinical hypothyroidism in this study, which used the trimester-specific reference range for TSH.

This study has a number of limitations. First, we only have measures of thyroid function at a single time point during and after pregnancy. However, this is not likely to have a significant impact on the conclusions of our study because it has been demonstrated that variations in an individual's thyroid function over a short duration tends to be narrow both in and outside pregnancy (16, 17). Furthermore, in routine practice, clinical decisions to treat subclinical hypothyroidism in pregnancy are normally made on a single measurement, which is in contrast to subclinical hypothyroidism diagnosed in a nonpregnant patient in whom thyroid function tests are generally repeated after a few weeks before treatment is started. With a single assessment of thyroid function 5 years after pregnancy, there may be changes at other time points not picked up by our analysis. However, our finding of normal thyroid function at this time in more than three quarters of women who had subclinical hypothyroidism during pregnancy still suggests that the majority of these women recover after pregnancy. Second, thyroid function tests were performed on stored serum samples. However, it has been shown that serum TSH, FT_4_, FT_3_, and TPO-Ab levels are stable even after prolonged storage (18). Finally, we used different cutoffs in and outside of pregnancy. Given that TSH levels are generally lower in pregnancy than outside of pregnancy, we could not use the same reference ranges for both. We chose to use trimester-specific cutoffs for TSH from the current guidelines in pregnancy.

In conclusion, we have provided evidence to suggest that most cases of subclinical hypothyroidism in pregnancy are transient, so treatment with l-thyroxine in these women may not be warranted outside of pregnancy. This result highlights the need for reassessment of the thyroid status of these women after pregnancy.

## References

[1] Vaidya B Hubalewska-Dydejczyk A Laurberg P Negro R Vermiglio F Poppe K. Treatment and screening of hypothyroidism in pregnancy: results of a European survey. Eur J Endocrinol. 2012;166:49–54 22023792 10.1530/EJE-11-0729

[2] Haddow JE McClain MR Palomaki GE Kloza EM Williams J. Screening for thyroid disorders during pregnancy: results of a survey in Maine. Am J Obstet Gynecol. 2006;194:471–474 16458648 10.1016/j.ajog.2005.07.055

[3] Blatt AJ Nakamoto JM Kaufman HW. National status of testing for hypothyroidism during pregnancy and postpartum. J Clin Endocrinol Metab. 2012;97:777–784 22170721 10.1210/jc.2011-2038

[4] Altomare M La Vignera S Asero P. High prevalence of thyroid dysfunction in pregnant women. J Endocrinol Invest. 2013;36:407–411 23095459 10.3275/8658

[5] Haddow JE Palomaki GE Allan WC. Maternal thyroid deficiency during pregnancy and subsequent neuropsychological development of the child. N Engl J Med. 1999;341:549–555 10451459 10.1056/NEJM199908193410801

[6] Krassas GE Poppe K Glinoer D. Thyroid function and human reproductive health. Endocr Rev. 2010;31:702–755 20573783 10.1210/er.2009-0041

[7] van den Boogaard E Vissenberg R Land JA. Significance of (sub)clinical thyroid dysfunction and thyroid autoimmunity before conception and in early pregnancy: a systematic review. Hum Reprod Update. 2011;17:605–619 21622978 10.1093/humupd/dmr024

[8] Pop VJ Kuijpens JL van Baar AL. Low maternal free thyroxine concentrations during early pregnancy are associated with impaired psychomotor development in infancy. Clin Endocrinol (Oxf). 1999;50:149–155 10396355 10.1046/j.1365-2265.1999.00639.x

[9] Finken MJ van Eijsden M Loomans EM Vrijkotte TG Rotteveel J. Maternal hypothyroxinemia in early pregnancy predicts reduced performance in reaction time tests in 5- to 6-year-old offspring. J Clin Endocrinol Metab. 2013;98:1417–1426 23408575 10.1210/jc.2012-3389

[10] Julvez J Alvarez-Pedrerol M Rebagliato M. Thyroxine levels during pregnancy in healthy women and early child neurodevelopment. Epidemiology. 2013;24:150–157 23232616 10.1097/EDE.0b013e318276ccd3

[11] Henrichs J Bongers-Schokking JJ Schenk JJ. Maternal thyroid function during early pregnancy and cognitive functioning in early childhood: the Generation R study. J Clin Endocrinol Metab. 2010;95:4227–4234 20534757 10.1210/jc.2010-0415

[12] De Groot L Abalovich M Alexander EK. Management of thyroid dysfunction during pregnancy and postpartum: an Endocrine Society clinical practice guideline. J Clin Endocrinol Metab. 2012;97:2543–2565 22869843 10.1210/jc.2011-2803

[13] Stagnaro-Green A Abalovich M Alexander E. Guidelines of the American Thyroid Association for the diagnosis and management of thyroid disease during pregnancy and postpartum. Thyroid. 2011;21:1081–1125 21787128 10.1089/thy.2011.0087 PMC3472679

[14] Garber JR Cobin RH Gharib H. Clinical practice guidelines for hypothyroidism in adults: cosponsored by the American Association of Clinical Endocrinologists and the American Thyroid Association. Endocr Pract. 2012;18:988–1028 23246686 10.4158/EP12280.GL

[15] Knight B Shields BM Hattersley AT. The Exeter Family Study of Childhood Health (EFSOCH): study protocol and methodology. Paediatr Perinat Epidemiol. 2006;20:172–179 16466435 10.1111/j.1365-3016.2006.00701.x

[16] Andersen S Pedersen KM Bruun NH Laurberg P. Narrow individual variations in serum T_4_ and T_3_ in normal subjects: a clue to the understanding of subclinical thyroid disease. J Clin Endocrinol Metab. 2002;87:1068–1072 11889165 10.1210/jcem.87.3.8165

[17] Boas M Forman JL Juul A. Narrow intra-individual variation of maternal thyroid function in pregnancy based on a longitudinal study on 132 women. Eur J Endocrinol. 2009;161:903–910 19773370 10.1530/EJE-09-0579

[18] Männistö T Surcel HM Bloigu A. The effect of freezing, thawing, and short- and long-term storage on serum thyrotropin, thyroid hormones, and thyroid autoantibodies: implications for analyzing samples stored in serum banks. Clin Chem. 2007;53:1986–1987 17954505 10.1373/clinchem.2007.091371

